# State of the Blood, Produced by the Absorption of Urine

**Published:** 1845-10

**Authors:** 


*State of the Blood, produced by Absorption of Urine.*—This form of the disease is described by Autenrieth and Schonlein, under the title of *urodialysis*, and by ancient authors, under that of *ischuria*, or *paralysis vesicalis*, *urpolania*, *&c.* It has been established by recent researches, that urea and uric acid are formed in the blood in consequence of defective renal secretion, and that it can be excreted from the blood by other organs than the kidneys. Urea has been found in the perspiration, the fæces, and the matters discharged by vomiting; in the air expired from the lungs, in the saliva, the tears, the milk, and the fluid exhaled from the serous membranes, the secretion from ulcers, &c.; and this has commonly happened with patients who had suffered for sometime with retention of urine. Numerous cases show that these vicarious secretions permit of retention of the urine existing for days, and even weeks, without danger to life.

The urea, when introduced into the blood, seems to produce a morbid condition of the whole stream, and thus destruction to life. For it is proved, that urea enters very readily into combinations, even by mere change of temperature, and that it is particularly inclined to transformation into carbonate of ammonia, while the latter salt possesses the property of readily dissolving the fibrin of the blood. The symptoms, described by modern authors under the title of *urodialysis senum*, are thus different from those observed by the author; for, in the latter, there was no pronounced acidity, no ischuria urinosa, asthma urinosum, nor prurigo semilis, &c. But they were very similar with those described by Morgagni and Stoll.—*Med. Times.*—*Braithwait's Retrospect.*

